# The origin of chow chows in the light of the East Asian breeds

**DOI:** 10.1186/s12864-017-3525-9

**Published:** 2017-02-16

**Authors:** Hechuan Yang, Guodong Wang, Meng Wang, Yaping Ma, Tingting Yin, Ruoxi Fan, Hong Wu, Li Zhong, David M. Irwin, Weiwei Zhai, Yaping Zhang

**Affiliations:** 1 Department of Molecular and Cell Biology, School of Life Sciences, University of Science and Technology of China, Hefei, 230026 China; 2 State Key Laboratory of Genetic Resources and Evolution, and Yunnan Laboratory of Molecular Biology of Domestic Animals, Kunming Institute of Zoology, Chinese Academy of Sciences, Kunming, 650223 China; 3 Human Genetics, Genome Institute of Singapore, A*STAR, 60 Biopolis Street, Genome #02-01, Singapore, 138672 Singapore; 4 Laboratory for Conservation and Utilization of Bio-resource & Key Laboratory for Microbial Resources of the Ministry of Education, Yunnan University, Kunming, 650091 China; 5 University of Chinese Academy of Sciences, Beijing, 100049 China; 6 Kunming College of Life Science, University of Chinese Academy of Sciences, Kunming, 650223 China; 7 Laboratory Medicine & Pathobiology, University of Toronto, 1 King’s College Circle, Rm 6211, Toronto, ON M5S 1A8 Canada

**Keywords:** Dog domestication, RAD sequencing, Demographic history, Artificial selection

## Abstract

**Background:**

East Asian dog breeds are one of the most ancient groups of dogs that radiated after the domestication of the dog and represent the most basal lineages of dog evolution. Among these, the Chow Chow is an ancient breed that embodies very distinct morphological and physiological features, such as sturdy build, dense coat, and blue/purple tongue.

**Results:**

Using a Restricted site Associated DNA (RAD) sequencing approach, we sequenced the genomes of nine Chow Chows from China. Combined with a dataset of 37 canid whole genome sequencing (WGS) from several published works, we found that the Chow Chow is one of the most basal lineages, which originated together with other East Asian breeds, such as the Shar-Pei and Akita. Demographic analysis found that Chow Chows originated from the Chinese indigenous dog about 8300 years ago. The bottleneck leading to Chow Chows was not strong and genetic migration between Chow Chows and other populations is low. Two classes of genes show strong evidence of positive selection along the Chow Chow lineage, namely genes related to metabolism and digestion as well as muscle/heart development and differentiation.

**Conclusions:**

Dog breeds from East Asia, including the Chow Chow, originated from Chinese indigenous dogs very early in time. The genetic bottleneck leading to Chow Chows and migrations with other populations are found to be quite mild. Our current study represents an early endeavor to characterize the origin of East Asian dog breeds and establishes an important reference point for understanding the origin of ancient breeds in Asia.

**Electronic supplementary material:**

The online version of this article (doi:10.1186/s12864-017-3525-9) contains supplementary material, which is available to authorized users.

## Background

Animal and plant domestication, one of the greatest innovations in recent human history, is a fundamental basis for modern civilization [1]. Of all the large mammals that are candidates for domestication (about 148 species with body weight greater than 90 lb), only a few species were successfully domesticated (14 species) [2]. Among these, the domestic dog (*Canis lupus familiaris*) is the only large carnivore that was able to thrive in a human-created environment [3]. Dog domestication represents one of the most enchanting evolutionary processes composed by human beings.

Even though, extensive efforts have been put into understanding the history of dog domestication, the conclusions are still greatly debated. For example, mtDNA, Y chromosome and whole genome sequencing (WGS) have pointed to southern East Asia as the region where dog originated from [4–8]. However, genetic comparisons between gray wolves and domestic dogs using SNP array suggested that the Middle East and Central Asia were important sources for dog domestication [9, 10]. Moreover, an ancient mtDNA work also suggested Europe as another site for the origin of dog domestication [11]. Thus, four geographic locations on the Eurasian continent have been suggested as the birthplace of dog domestication and the origin of dogs seem to be a great mystery in the light of these different studies.

Although multiple centers have been proposed for dog domestication, the indigenous dogs from China and several ancient breeds from East Asia embody the highest amounts of genetic variability and are identified as the basal lineages after arising from gray wolves [4–8, 12]. There are several endemic classes of breeds in East Asia, including working dogs (Tibetan Mastiff, Akita, Samoyed and Siberian Husky), toy dogs (Pekingese, Pug, Shih Tzu and Japanese Chin) and various other breeds with very diverse temperament and appearance (Chow Chow, Shar-Pei, Lhase Aspo, Shiba Inu and Jindo) [13]. Compared with many European breeds, which were selected recently in the past few hundred years through intense artificial selection [13], East Asian ancient breeds tend to carry substantially more genetic variability and are morphologically as distinctive as the European breeds [14]. Given the wide geographic distribution of Chinese indigenous dogs across the country side of China (see Wang et al. for a description of Chinese indigenous dogs [8]), how East Asian dogs originated and evolved from each other is an interesting question awaiting answers.

One of the most interesting ancient breeds in East Asia is the Chow Chow. In Chinese history, the Chow Chow often appears as a symbol similar to the traditional stone guardians (stone lions) found in front of Buddhist temples and palaces. It has a sturdy build with a very dense coat, particularly thick in the neck area. In addition, Chow Chows also have several distinguishing features including an extra pair of teeth (44 instead of 42), an unusual blue-black/purple tongue and straight hind legs, resulting in a rather stilted gait. In this study, we conducted Restricted site Associated DNA (RAD) sequencing [15] on nine Chow Chows sampled from China. Combining these sequences with WGS data from many other dogs and relatives, we inferred the origin of the Chow Chow in light of the East Asian dogs and identified adaptively evolving genes along the Chow Chow lineage.

## Methods

### Sample collection and RAD sequencing experiment

We collected blood samples from nine Chow Chows, three of which were collected in Beijing and the other six from Kunming. In order to balance sequencing cost and the number of individuals we can study, we chose the RAD sequencing approach to survey the Chow Chow genomes. After simulating the cut sites in the dog reference genome Canfam3.0 using all type II restriction enzyme from REBASE [16], we selected SpeI (a six mer, A^CTAGT), which has 340,847 predicted cutting sites across the dog reference genome. After extracting the genomic DNA using QIAamp DNA Blood Mini Kit from QIAGEN, SpeI was used to incubate the genomic DNA for 16 h. The resulting short fragments were ligated to the sequencing adaptor primers (P1). Subsequently, DNA fragments were sonicated to shorten the fragments. Following size selection using electrophoresis, we used NEBNext Ultra DNA Library Prep Kit from Illumina to repair the fragments and ligate the Y adapters to the sonicated fragments. Paired PCR primers with one complementary to the P1 adaptor and the other containing both the barcode as well as the complementary sequence to one arm of the Y adaptor were used to amplify the target genomic segments, where one end has the P1 adaptor and the other end has a Y adaptor. After PCR amplification, the resulting library was quantified using an Agilent 2100 bioanalyzer. Equal amount of DNA were subsequently pooled for sequencing using the Hiseq 2000 platform at the Kunming Institute of Zoology.

### Public data curation

Sets of WGS data were collected from four previous studies. The first study [7] included four gray wolves, three Chinese indigenous dogs and three dog breeds (Tibetan Mastiff, Belgian Malinois, German Shepherd). We collected six breed dogs from the second study [17]. They are one Afghan Hound, one Labrador Retriever, one Chow Chow, one Tornjack (Croatian Shepherd Dog), one Istrian Shorthaired Hound and one Caucasian Ovcharka. Genome sequences from 10 Tibetan Mastiffs and 10 Chinese indigenous dogs from Yingjiang (Yunnan, China) were obtained from the third study [18]. We also collected data for one Jindo dog from Korea published in 2012 [19]. In total, we collected genome sequences from 37 canids, which included four gray wolves, 13 Chinese indigenous dogs, 11 Tibetan Mastiffs, one Chow Chow and eight other dog breeds (Additional file 1: Table S1). In addition to the sequencing data, we also used a SNP array dataset, which contains about 48,000 SNPs from 1191 canids [20].

### Read mapping and variant calling

After downloading the WGS data from the NCBI/DDBJ SRA repository (Additional file 1: Table S1), we mapped the short reads as well as our RAD sequencing data to the reference genome (Canfam3.0) using BWA (version 0.6.2-r126) [21]. Picard (version 1.87) [22] was used to mark duplications and GATK (version 2.7-2-g6bda569) [23] was used to perform base recalibration and local realignment. BAM files from both the WGS and RAD sequencing were conjugated to call variants jointly using mpileup in SAMtools package (version 0.1.19-44428 cd) [24]. Subsequently, Perl script vcfutils.pl in SAMtools package was used to extract the high fidelity variants for the downstream analysis.

### Experimental verification

We randomly selected 14 E1 regions (from read1 that is adjacent to the restriction enzyme cutting site) and 11 SNPs from outside E1 regions to validate our SNP set. We used Sanger sequencing for all of those regions in eight Chow Chows. In order to calculate false positive and false negative for the SNP calling, we first identify genomic regions of each Sanger reads. SNPs found in Sanger, but not in the SNP set is designated as false negative. SNPs private to the SNP set, but not found in the Sanger sequencing is designated as the false positive.

### Genetic diversity, kinship, principle component analysis and population structure analysis

Heterozygous sites within each individual were used to calculate the genetic diversity. For the WGS data, genetic diversity was calculated as the percentage of heterozygous sites within each window. For the RAD data, due to the non-uniform coverage across the genome, we exclude sites where coverage was less than five times and the genetic diversity was calculated focusing solely on the remaining sites. We used a window size of 1 Mb and step size of 200 kb.

We used the software KING [25] to investigate the relationship between nine Chow Chows sequenced in this study. As a comparison, we also computed the kinship between Chow Chows (12 individuals) from the SNP array data [20].

We combined all the sequencing data (RAD and WGS) with a SNP array dataset [20], and then performed principle component analysis (PCA) and population structure analysis over the combined datasets. PCA was carried out using smartPCA in EIGENSOFT (version 4.2) [26]. Population structure analysis was carried out using ADMIXTURE (Version 1.23) [27] and the results of the population structure analysis were plotted using CLUMPAK (Version 1.1) [28].

### Linkage disequilibrium

In order to compare linkage disequilibrium (LD) across different dog and gray wolf populations, we combined the WGS data with the array data [20] and extracted 45,766 SNPs, which are genotyped in both datasets. Subsequently, we selected populations that had at least nine individuals. After randomly selecting nine individuals from each of these populations, linkage disequilibrium in terms of correlation coefficient (r-square) was calculated between all sites whose distances between each other is less or equal to 500 kb using PLINK (Version 1.07) [29]. After processing each population, we binned distances into discrete 5 kb windows (500 kb/5 kb = 100) and calculated the mean r-square for all windows of different sizes. In order to measure the overall level of linkage disequilibrium, we defined a H statistic which is the sum of all the LD values for different 5 kb windows (from 5 kb to 500 kb at step of 5 kb). In other words, H statistic is an analog of the area under the curve for mean LD across 500 kb windows and captures overall levels of LD within each population.

### TreeMix and the three-population test

In order to focus our analyses on the East Asian breeds, we combined all samples that were sequenced by WGS in our collection with a set of East Asian breeds (those that locate in the group 1 cluster in the PCA analysis of all samples) and the Samoyed, which has been thought to be a potential ancestor population for the Chow Chow due to morphological similarities between the two breeds [30], from the SNP array data. Since Chow Chows from different sources are quite concordant, we choose to use the Chow Chows from the SNP array as the representative set to do this inference (combining the SNP array with the RAD data will leave us with too few SNPs for the TreeMix analysis). TreeMix (Version 1.1) [31] was used to perform the analysis. The three-population test was conducted using ADMIXTOOLS (Version 1.1) [32] across all population combinations.

### Demographic inference

We used G-PhoCS (Version 1.2.2) [33] to infer the demography history of Chow Chows together with gray wolves and Chinese indigenous dogs. First of all, we used a series of filters to select independently evolving neutral loci across the genome. For most SpeI cutting sites, both the upstream and downstream regions adjacent to them will be sequenced. We selected all the SpeI cut sites with high coverage (i.e. 100 bp flanking regions on both sides were sequenced at least five times in each individual among the nine RAD sequenced Chow Chows). The 100 bp sequences flanking the cutting sites were joined together (with the sequence motif ‘ACTAGT’) and 3 bp subsequently trimmed from both ends of the sequences (bases at these ends tend to have lower quality scores), producing 200 bp loci at each restriction enzyme cutting site. All the extracted loci overlapping with CpG islands, repeat regions, gap regions in Canfm3.0 were also removed (annotation was downloaded from UCSC genome browser [34]). In order to focus our analysis on the neutrally evolving regions of the genome, we retained all the sequences at least 10 kb distance from exons and more than 100 bp away from conserved noncoding elements (CNEs). We used the gene annotation information from both UCSC [34] and NCBI database [35]. We extracted the CNE information of dog genome similar to the method described in a previous study [36], using an updated dataset of the multi-species alignment located at UCSC [37]. Since G-PhoCS requires independently evolving loci across the genome, we took one locus every 100 kb. These filters identified 13,468 regions, each with 200 bp in length, which could be used for the demographic inference.

We extracted sequences from all these 13,468 regions from all four gray wolves, eight Chinese indigenous dogs (subgroup 1) and all 10 Chow Chows. We randomly selected 2000 loci across the genome and picked four individuals from each population to perform the demographic inference. The Markov Chains were run for 5,200,000 iterations, with the first 200,000 iterations treated as burn-in. Chains were sampled every 10 iterations. We randomly subsampled five replicate datasets and results from these randomly selected datasets were then combined for the final result.

### Mutation rate calibration

Mutation rate is a very important parameter for the demographic inference. Using multiple species as the outgroup to the canids, we calibrated the mutation rate along the dog lineage using neutral sequences across the genome similar to one of the recent study [8] (Additional file 2: Note S1). To translate the results into real units, we used this calibrated mutation rate from comparative genomic analysis (2.2*10^−9^ per site per year) and a generation time of 3 years [7, 8, 38].

### Selective sweeps and Gene Ontology analysis

We used both population branch statistic (PBS) [39] and SweepFinder [40] to identify the selective sweep regions in the genomes of Chow Chow. For PBS, pairwise window-based Fst values (window size 100 kb and step size 20 kb) were calculated using VCFtools (Version: 0.1.11) [41] among the Chinese indigenous dogs, Tibetan Mastiff and Chow Chows. The PBS for the Chow Chows was calculated as (T_CI_ + T_CT_-T_IT_)/2 [39]. T was computed as -log(1-*F*_*ST*_) and the subscripts C stands for the Chow Chow, I stands for the Chinese indigenous dogs and T stands for the Tibetan Mastiff. Higher PBS values represent long evolutionary distances in terms of allele frequency differences along the Chow Chow lineage.

For the SweepFinder, we used a dhole’s genome as the outgroup [7] to identify the ancestral states for all of the SNP positions. Then, using the genome background site frequency spectra as a control (all of the autosomal chromosomes), we employed SweepFinder [40] as an independent approach to identify traces of selective sweeps.

We selected the intersection of the top 3% of the PBS regions and the top 3% of the SweepFinder regions as the candidate regions for the final set. Gene annotation was based on the Ensembl annotation [42]. We then converted those dog gene IDs to their associated human gene IDs using ensemble homologous mapping extracted from Ensembl BioMart portal [42]. Gene Ontology (GO) analysis was conducted using DAVID [43].

In order to investigate the genetic basis of supernumerary teeth in Chow Chows, we conducted literature survey and found that four important pathways (BMP, FGF, SHH and WNT) are involved in the teeth development [44]. Genes associated with these pathways were extracted from WikiPathways [45] (for the BMP, SHH and WNT pathway) and the literature [46] (for the FGF pathway). In addition, we also curated from the literature a list of genes causing tooth abnormalities in transgenic mice [44]. These genes were combined and used as the list of candidates responsible for the teeth development. We subsequently overlapped the list of selected genes with this gene list, looking for possible candidate genes responsible for the different number of teeth in Chow Chows. For the genetic basis of the blue tongue of Chow Chows, we extracted all pigmentation genes from Color Genes database [47].

## Results

### Sample collection

Using a modified RAD construction protocol [15], sequencing libraries from nine individuals were pooled and sequenced using the Illumina platform. In RAD sequencing, one end of the paired-end sequencing (denoted as E1) is strictly positioned at the same restriction cutting site and has uniform coverage, while the other end (denoted as E2) is variable in position depending on the insert size. On average, each individual is sequenced to about 36-fold at the E1 site (Additional file 1: Table S1) and the E1 reads cover about 2.7% of the whole genome.

**Fig. 1:**
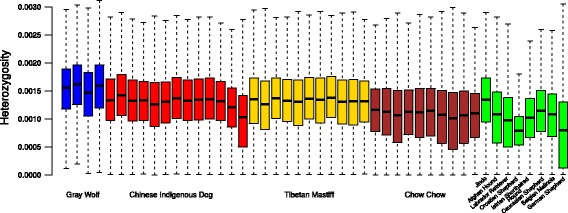
Genetic diversity (Heterozygosity) across 46 canids. Boxplot for heterozygosity across 46 canids were plotted in this graph. The middle line represents the median, and the box represents the interquartile range; bars extend to 1.5 times the interquartile range. The color corresponds to different population groupings

The sequence data generated by our RAD sequencing was combined with a dataset consisting of whole genome sequences for 37 canids curated from four published studies [7, 17–19] (Additional file 1: Table S1, Fig. 1). In total, we called 16,716,649 SNPs across the whole genome (not limited to the RAD regions). The transition/transversion ratio of this set is 2.186, indicating good quality results from the variant calling procedure implemented in SAMtools [48]. We denote this SNP set as the whole genome SNP set (i.e. WG SNP set). Since the sequence coverage from the RAD sequencing will be restricted to certain genomic regions, we further filter the SNP set by targeting on the genomic regions with good coverage from the RAD individuals (i.e. genotype quality > =20 in at least six out of nine individuals) and extracted 1,130,910 high quality SNPs (denoted as RAD SNP set). Using Sanger validation, we found that, the false positive and false negatives in variant calling in the RAD data are 5.2 and 6.4%, respectively (Additional file 1: Table S2). The subsequent population genetic analysis was conducted using different combination of these two SNP sets (Additional file 1: Table S3). Using a kinship estimation procedure [25], we found that Chow Chows in our collection are not closely related and show similar kinship value to the individuals from the SNP array (Additional file 3: Figure S1).

### Genetic diversity across the genome

Using heterozygous SNPs called from each individual, we calculated the genetic diversity for each individual across the genome. In Fig. 1, we plotted the genome wide distribution of variation for all 46 individuals. As we can see, there is a general trend of decreasing diversity from gray wolves to Chinese indigenous dogs, Tibetan Mastiff and Jindo. Chow Chow together with many other dog breeds, most of which are from the Middle East and Europe have lower genetic diversity.

Among dog breeds, genetic diversity varies quite extensively. For example, most of the dog breeds from outside East Asia have genetic diversity similar to the Chow Chow. The only two exceptions are shepherd dogs from Germany and Croatia which possess much reduced genetic diversity. Among the East Asian dogs, the Chinese indigenous dogs, Tibetan Mastiff and Jindo have comparable levels of diversity, which are higher than the diversity of the Chow Chow.

### Principle component analysis

**Fig. 2:**
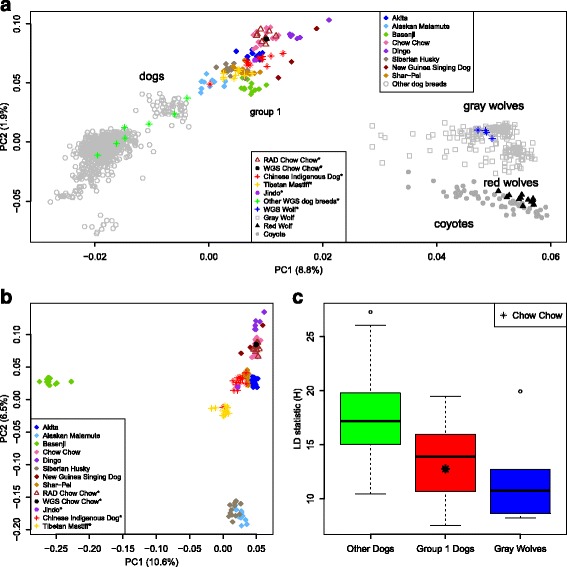
Principle component analysis of canids and linkage disequilibrium across populations. **a** PCA results for the first two PCs were plotted here for 1237 canids. The percentage of variances explained by the two PCs are also shown. Different symbols corresponding to different populations are shown in the legend (* marks sequenced samples in this study). **b** PCA analysis of group 1 dogs. The percentage of variances explained by the PCs and the symbols are shown similar to panel **a**. In the legend, * marks sequenced samples in this work. **c** Linkage disequilibrium of the gray wolves, group 1 dogs as well as other dogs are plotted as boxplots. Six wolf populations, eight group 1 dog populations as well as 66 other dog populations are shown in this figure. Chow Chow is shown as an *asterisk*. The Y axis (the H statistic) is the numerical measurement of linkage disequilibrium across a 500 kb window (see Methods)

To explore the genetic relationships among these dogs, we combined the RAD SNP set of the 46 individuals (37 WGS samples and nine RAD sequenced Chow Chows) with a previously published SNP array dataset [20] and performed a principle component analysis (PCA) on this combined dataset. In Fig. 2a, we plotted the relationships of these samples. The first PC, which accounts for 8.8% of the total variation, separates the dogs from the gray wolves and other canids.

Across all worldwide dog populations, we can clearly see a wide distribution in the genetic differences, including a dense cluster of individuals that are much closer to the gray wolves than to other dogs (denoted as group 1, Fig. 2a). This dense cluster includes a large group of East Asian breeds and a few other breeds from other geographic locations. For example, New Guinea Singing Dog and Dingo are currently from Australia, but are found to have spread from south East Asia [49]. The Alaskan Malamute is from Alaska, which is close to East Asia. The only exception is the Basenji, which is an African dog breed that previously was found to have admixture from the gray wolf [31]. Quite reassuringly, multiple sources of Chow Chows are concordant with each other in the PCA plot. This suggests that the sample quality among the diverse sets of data collected for the Chow Chow is very consistent.

To further dissect the relationships among the group 1 individuals, PCA analysis was conducted among these individuals (Fig. 2b). We found that the clustering pattern correlates quite well with the geographic origin of these individuals. For example, the Basenji stays distinct from the remaining individuals along the first axis (PC1). Subsequently, breeds from the arctic regions (Siberian Husky, Alaskan Malamute) are separated from the others along the PC2 axis. The rest of the East Asian breeds stay close to each other.

Within the core East Asian cluster (Fig. 2b), there are still different degrees of closeness. Running from the top of the PCA plot are a) Dingo and New Guinea Singing dog, b) Chow Chow, c) Shar-Pei, Akita, Chinese indigenous dogs and Jindo, and d) Tibetan Mastiff. Among the Chinese indigenous dogs, the distribution is rather heterogeneous and they are quite scattered across the genetic landscape (also see later sections). Genetically, the Chow Chow is slightly differentiated from the other East Asian breeds (e.g. Shar-Pei, Akita, Chinese indigenous dogs, Jindo), which could be related to their unique history of origin.

When we examined the extent of linkage disequilibrium (LD) across the groups, especially using a numerical measurement (denoted as the H statistic) to capture the overall extent of linkage disequilibrium for each population, we found that group 1 dogs have a greatly reduced level of linkage disequilibrium compared to other dog breeds (Fig. 2c). Interestingly, the LD level of the Chow Chow is fairly low compared with other group 1 breeds. The low LD in the Chow Chow suggests its ancient origin or a relatively mild bottleneck at the time of origin.

### Population structure analysis

**Fig. 3:**
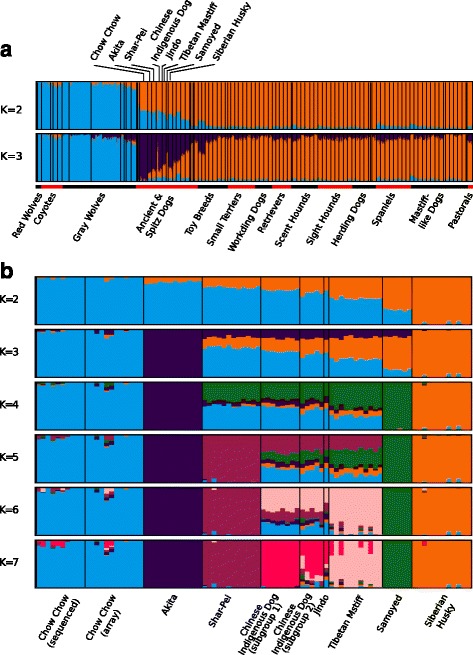
Structure analysis of the canids. **a** Structure analysis of all the canid data for K = 2-3 (Populations with only one sample are not included for this analysis except the Jindo). The groups of different dog types were extracted from Vondolt et al. [9]. The focal group 1 populations are marked on top of the panel. **b** Structure analysis of all the East Asian breeds with different number of groups (K = 2-7). Colors mark different groupings from the structure analysis

Population structure analysis provides a powerful alternative approach for exploring the relationships among multiple individuals. When combining the sequenced East Asian dogs with a large number of canids from the SNP array collection [20], we see that the East Asian individuals are the ones most similar to the gray wolves (Fig. 3a), matching our observation from the PCA plot (Fig. 2a). To further explore the genetic relationships among the East Asian lineages, we conducted a structure analysis restricted only to the East Asian breeds (Fig. 3b). When partitioning the set into two groups, Chow Chow and Siberian Husky are the two extremes of the landscape (Fig. 3b). The other populations are intermediates between these two groups, which matches the earlier PCA analysis (Fig. 2b). Further partitioning the set into more groups, leads to the separation of the Akita (K = 3), Samoyed (K = 4), Shar-Pei (K = 5), Tibetan Mastiff (K = 6), and the Chinese indigenous dogs (K = 7), which contain a subset of mixed constituent individuals [8]. The Korean breed Jindo shows a similar profile to the Chinese indigenous dogs, matching the earlier results from the PCA analysis (Fig. 2b).

We found two distinct subgroups in the Chinese indigenous dogs. One group (denoted as subgroup 1) has relatively pure genetic constitutions and the other subgroup (denoted as subgroup 2) from northern China tends to have more mixed genetic components. This also agrees with the earlier observation that the Chinese indigenous dogs show a wide range of distributions across the PCA plot (Fig. 2a and b).

### TreeMix analysis

**Fig. 4:**
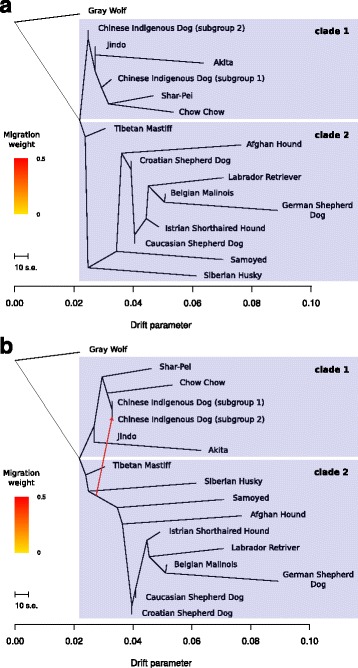
TreeMix analysis of the East Asian breeds together with our WGS collection. **a** TreeMix results for the analysis without allowing for any migration track. The x-axis corresponds to the amount of genetic drift. Clade 1 is all from East Asia while clade 2 has a mixture of East Asian breeds and non-Asian breeds. **b** TreeMix results for the analysis allowing one migration track. The inferred migration track is shown as a red arrow in the phylogenetic tree. The weight of migration component is scaled according to the rainbow in the left

The population structure and PCA analyses allowed us to explore the genetic closeness of these groups, but it does not provide detailed evolutionary relationships among these populations. To explore the phylogenetic relationship among these individuals, we conducted a TreeMix analysis [31] (Fig. 4 and Additional file 4: Figure S2) of all the populations suggested by the structure analysis. Given that we are particularly interested in the East Asian breeds, we combined the WGS dataset of 37 individuals with a few East Asian breeds from the SNP array study.

In Fig. 4a, we see that there are two deeply divergent lineages among the dogs (denoted as clade 1 and 2, Fig. 4). One clade represents a subgroup of breeds from East Asia while the other clade includes the Tibetan Mastiff, Arctic groups and many of the other Breeds from the Eurasian continent (Fig. 4a). It is interesting to observe that, the Tibetan Mastiff clustered at the basal position of the two clades and grouped with the non-Asia clade (clade 2). Surprisingly, the two subgroups (subgroup 1 and 2) of indigenous dogs informed by the structure analysis were separated in the TreeMix analysis.

Using the three-population test implemented in ADMIXTOOLS [32], we tested for the possibility of admixture events across all of these populations. We found that only subgroup 2 of the Chinese indigenous dogs bear a strong signal of admixture, and that the two source populations contributing to the admixture are always one population from clade 1 and one from clade 2 (Additional file 1: Table S4).

In light of the three-population test results, we allowed one migration track in the TreeMix analysis (Fig. 4b). We see that the two subgroups of Chinese indigenous dogs are now clustered together and that a migration track from clade 2 contributed a source component for the tentatively admixed subgroup in the Chinese indigenous dogs (subgroup 2). There seems to be at least two subgroups in the Chinese indigenous dogs, where one subgroup is pure in genetic constitution, while the other bears some migratory/admixture signal from the clade 2 lineages [8]. In addition, Chow Chows are the breed that is closest to the Chinese indigenous dog comparing to other Asian breeds.

### Demographic inference

**Fig. 5:**
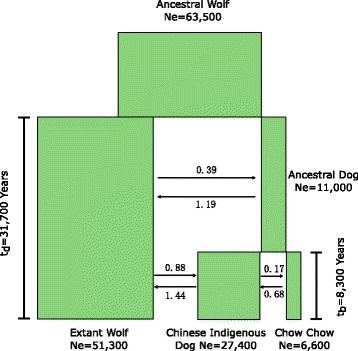
Demographic model for the origin of the Chow Chow. The results from the G-PhoCS analysis are depicted in this figure. Divergence time (in years), population size and migration rate (2 Nm) were shown together with the demographic history

As an ancient breed originated in China, the time and process that gave rise to the Chow Chow should be informative for our understanding of breed formation in East Asia. Using a Markov Chain Monte Carlo approach based on the divergence between the multiple sequences [33] and a well calibrated mutation rate (Additional file 2: Note S1, Additional file 1: Table S5), we dated the origin of the Chow Chow from the Chinese indigenous dogs (Fig. 5 and Additional file 1: Table S6). We found that dogs separated from the gray wolves in East Asia about 31,700 years ago, matching several earlier discoveries [7, 8]. After the separation of these two populations, the ancestral Chinese indigenous dogs maintain a relatively small population. The time of origin for the Chow Chow from the Chinese indigenous dogs was estimated to be 8300 years ago. Interestingly, the population size of the Chinese indigenous dogs increased quite rapidly after the split from the Chow Chow, while the Chow Chow’s overall population size show a slight decrease in size comparing to the population size of the ancestral Chinese indigenous dogs. The levels of migration estimated between the wolves and dogs, as well as among dog groups are quite low (2 Nm ~1 or lower). This suggests that the East Asian lineages stayed relatively distinct from each other during the history of dog evolution.

### Candidates for artificial selection

**Fig. 6:**
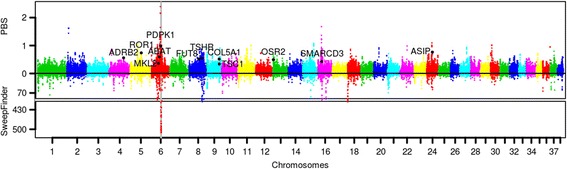
Genome wide evidence for selection using both PBS and SweepFinder. A Manhattan plot across different chromosomes for the evidence of positive selection. The top panel is for the PBS statistic and the bottom panel is for the SweepFinder. Shadowed area is the region on chromosome 6 with the strongest signal for selection. A selected gene list is marked on the genome wide plot

Given the Chow Chow’s unique morphological and physiological features such as sturdy build, dense coat, supernumerary teeth and blue/purple tongue, we wanted to understand the genetic basis of these traits, especially whether there are traces of positive selection at the loci responsible for these interesting phenotypes. We used both an Fst based method PBS [39] and a composite likelihood method SweepFinder [40] to scan the Chow Chow genomes for traces of recent adaptation. In order to be conservative in our discoveries, we required that the candidate regions be within the top 3% for both measurements. After annotating these regions (0.81% of the genome), we identified 226 genes with strong signal for adaptive evolution along the Chow Chow lineage (Fig. 6).

Classifying these genes using Gene Ontology (GO) [43], we found that there are two major functional categories that were selected along the Chow Chow lineage after separating from the Chinese indigenous dog. The first class of genes is related to digestion and metabolism (Table 1 and Additional file 1: Table S7). Genes involved in multiple amino acid digesting processes (e.g. Proline and Glutamine) as well as lipid metabolism are strongly enriched in this first class. It is quite interesting to observe this, since strong selection in genes involved in metabolism and digestion was also observed along the Chinese indigenous dog lineage [7, 8]. This might suggest that digestion and metabolism are fundamental to the evolution of dogs, and are constantly being tuned for their new diets in a rapid paced human environment.

**Table 1:** Gene Ontology (GO) analysis of positively selected genes in Chow Chow

GO terms	*P* Value	Enrichment fold	Genes mentioned in the main text
Proline biosynthetic process	0.002	42.946	-
Proline metabolic process	0.004	30.062	-
Adult behavior	0.01	5.826	*ASIP, TSHR, ABAT*
Glutamine family amino acid biosynthetic process	0.015	15.822	-
Oxidation reduction	0.025	2.039	-
Lysosomal transport	0.025	12.025	-
Intracellular transport	0.03	1.983	-
Feeding behavior	0.031	5.809	*ASIP*
Muscle cell differentiation	0.032	4.141	*SMARCD3, TSC1, MKL2*
Heart morphogenesis	0.036	5.491	*SMARCD3, COL5A1, MKL2*
Positive regulation of striated muscle development	0.039	50.104	*ADRB2, MKL2*
Positive regulation of muscle development	0.039	50.104	*ADRB2, MKL2*
Vacuolar transport	0.04	9.394	-
Lipid biosynthetic process	0.042	2.482	-
Respiratory gaseous exchange	0.049	8.351	*FUT8*
PcG protein complex	0.005	28.404	-
Pyrroline-5-carboxylate reductase activity	0.001	55.247	-
Oxidoreductase activity, acting on the CH-NH group of donors, NAD or NADP as acceptor	0.016	15.346	-
Oxidoreductase activity, acting on the CH-NH group of donors	0.039	9.525	-
Steroid dehydrogenase activity	0.044	8.911	-
Hsa00150:Androgen and estrogen metabolism	0.005	10.779	-

The second class of genes is related to muscle and heart development (Table 1 and Additional file 1: Table S7). For example, multiple GO terms, ranging from muscle cell differentiation to multiple categories of muscle development, are associated with this class of genes. Among the genes related to muscle development, *SMARCD3* is particularly interesting and is expressed specifically in the heart and somites in the early mouse embryo. Experimental silencing of this gene in mice using RNA interference resulted in abnormal cardiac and skeletal muscle differentiation [50]. In humans, the protein product of this gene jointly acts with the muscle determination factor MyoD to reprogram hESCs into skeletal muscle cells [51].

In addition, multiple other genes related to muscle and heart development such as *TSC1* [52], *MKL2* [53], *ADRB2* [54] and *COL5A1* [55] also show evidence of adaptive evolution (Additional file 1: Table S8).

Other than muscle development, adaptive evolution of genes involved in biological processes such as respiratory gaseous exchange and adult behavior are also quite interesting (Table 1 and Additional file 1: Table S7). *FUT8* encodes an enzyme belonging to the family of fucosyltransferases, and homozygous deletion of this gene in mouse shows emphysema-like changes in the lung [56]. *ABAT* is a gene responsible for the catabolism of an important neurotransmitter gamma-amino butyric acid (GABA). *TSHR* is an important gene with multiple functions including the regulation of metabolism and the photoperiod control of reproduction in vertebrates. This gene has been found to be selected across many other domesticated species including cat [57], chicken [58] as well as sheep [59].

In order to look into the possible genetic underpinnings of teeth developments (Chow Chows have 44 teeth instead of 42), we collected all the genes involved in teeth development. Overlapping these genes and the list of 226 positively selected genes yielded two candidate genes *OSR2* and *ROR1. ROR1* (homologous to another receptor tyrosine kinase *ROR2*) was a member of the WNT signaling pathway. Both *Ror1* and *Ror2* genes expressed in molar tooth primordia in mouse [60], and *Ror2*(−/−) mice exhibited defective differentiation of tooth [61]. Mice lacking *Osr2* gene developed supernumerary teeth lingual to their molars [62].

## Discussion

Using the Chow Chow as an example breed, we have made a systematic study of an ancient East Asian dog breed. There are several interesting observations that are worth discussing here. First of all, the time of origin of the East Asian breeds is perhaps quite old. For example, in the phylogenetic relationship presented in Fig. 4, the Chow Chow is the breed closest to the Chinese indigenous dogs. If all other dog breeds also begin from the Chinese indigenous dogs, then they all must have originated even earlier than 8300 years ago (Fig. 4). The mild population bottleneck leading to the Chow Chow suggests a gradual process leading to this breed. Historically, based on the morphological feature of the dense coat, Chow Chow has often been thought of a breed of high latitude origin [58]. However, the analysis here showed that, Chow Chow was selected from Chinese indigenous dogs, which are of Southern origin [8]. Given the fact that agriculture started in East Asia around 11,000 to 9000 years ago near the Yangtze River [63], the sedentary environment of humans could have facilitated the selection of the Chow Chows from Chinese indigenous dogs. Studying the historical context of the East Asian breeds together with modern human development is an enchanting picture waiting to be unveiled.

Secondly, even though the arise of Chow Chows has been hypothesized to be gradual, the amount of overall gene flow found between the Chinese indigenous dogs and Chow Chows is surprisingly low. It is possible that when an incipient breed is under development, the amount of genetic exchange between the source population and the population of interest could be quite high, as the amount of differentiation is still low [64]. However, the inference results show the opposite pattern (low migration). This suggests that the creation of Chow Chows can be very fast and subsequent interbreeding was restricted (possibly disfavored by human beings or there were behavior differences between breeds). Given the overall low migration rate found among East Asian breeds, this mode of breed formation might be quite general across many ancient breeds.

Thirdly, our gene-based analysis using gene ontology matches only a subset of the expectations from the phenotypes that are unique to Chow Chow. For example, the blue tongue and thick coats are not strongly indicated in the GO analysis results. A possible explanation for this is that the genes responsible for these phenotypes can be quite simple (only limited to a few genes) and cannot be easily be picked up by the GO based analysis. Very interestingly, we found a large region on chromosome 6 that showed a very strong signal for selection (around 7 Mb, Fig. 6). Inspecting all genes in this region with a database of pigmentation genes [47] found one gene *PDPK1*, which encodes 3-phosphoinositide dependent protein kinase 1. Mutations in *PDPK1* cause abnormal pigmentation in mouse embryos [65]. The origin of this large sweep region should be worth pursuing in future studies. Another gene related with pigmentation on our list of selected genes is *ASIP*, which is located on chromosome 24. It affects the pigmentation phenotype in many different animals [66–70].

Extensive selection in genes involved in muscle and heart development as well in adult behavior is consistent with the fact that the Chow Chow was kept as a sporting dog during its early development [58]. Interestingly, other animals such as horses also show evidence of strong positive selection in genes involved with muscle and cardiac development [71]. Future studies in other sport dogs should unveil a more dynamic picture of positive selection, some of which might be quite similar to that seen in the Chow Chow.

Lastly, using multiple public datasets, we revealed the existence of two subgroups of dogs within the Chinese indigenous dogs. One group is rather pure in terms of the genetic makeup while the second group shows admixture between the East Asian lineage and the non-Asian lineage (clade 2), matching the finding from a recent study [8]. Given the fact that most of the samples were taken from the southern part of China, understanding the genetic makeup of the Chinese indigenous dogs across China and East Asia will be quite important for our understanding of the origin of dogs in relation to these other lineages.

## Conclusions

Using RAD sequences from nine Chow Chows together with whole genome sequences from 37 canids, we characterized the origin of this ancient breed. Demographic inferences found that, Chow Chows originated from Chinese indigenous dogs 8300 years ago. The evolutionary process leading to the Chow Chow is accompanied by low levels of gene flow and mild population bottleneck. Two classes of genes showed strong evidence of positive selection along the Chow Chow lineage, namely genes related to metabolism and digestion and those related to muscle/heart development and differentiation. The study of Chow Chows offered an important insight into the history and process giving rise to East Asian breeds.

## Additional files

Additional file 1:
**Table S1.** Sample information. **Table S2.** Sanger sequencing validation in SNP calling. **Table S3.** SNP sets used in different analyses. **Table S4.** The three-population test result for the Chinese indigenous dogs (subgroup 2). **Table S5.** Mutation rate estimation. **Table S6.** G-PhoCS estimation of the demographic history. **Table S7.** Gene Ontology (GO) analysis of the positively selected genes in Chow Chows. **Table S8.** The list of selected genes with highlighted functions. (XLSX 27 kb)
Additional file 2: Note S1. Mutation Rate Estimation. (DOCX 44 kb)
Additional file 3: Figure S1. Boxplot of the kinship coefficient between pairs of Chow Chows in the RAD set and from the SNP array data. Different levels of relatedness will yield different kinship coefficients. For example, it is suggested that the estimated kinship coefficient range [0.354, 1], [0.177, 0.354], [0.0884, 0.177] and [0.0442, 0.0884] correspond to duplicate/monozygotic twin, 1st-degree, 2nd-degree, and 3rd-degree relationships respectively. All kinship coefficients from the RAD sequenced Chow Chows are smaller than 0.0442, and are comparable to the Chow Chows from the SNP array data. (PDF 4 kb)
Additional file 4: Figure S2. Residual of the TreeMix analysis presented in Fig. 4. Panel A and B correspond to the panels in the Fig. 4. (PDF 116 kb)

## References

[1] Diamond J (2002). Evolution, consequences and future of plant and animal domestication. Nature.

[2] Diamond J. Guns, Germs, and Steel: The Fates of Human Societies. New York: W. W. Norton & Company; 2005.

[3] Coppinger R, Coppinger L. Dogs: A Startling New Understanding of Canine Origin, Behavior & Evolution. New York: Scribner; 2001.

[4] Ding ZL, Oskarsson M, Ardalan A, Angleby H, Dahlgren LG, Tepeli C, Kirkness E, Savolainen P, Zhang YP (2012). Origins of domestic dog in southern East Asia is supported by analysis of Y-chromosome DNA. Heredity.

[5] Pang JF, Kluetsch C, Zou XJ, Zhang AB, Luo LY, Angleby H, Ardalan A, Ekstrom C, Skollermo A, Lundeberg J (2009). mtDNA data indicate a single origin for dogs south of Yangtze River, less than 16,300 years ago, from numerous wolves. Mol Biol Evol.

[6] Savolainen P, Zhang YP, Luo J, Lundeberg J, Leitner T (2002). Genetic evidence for an East Asian origin of domestic dogs. Science.

[7] Wang GD, Zhai W, Yang HC, Fan RX, Cao X, Zhong L, Wang L, Liu F, Wu H, Cheng LG (2013). The genomics of selection in dogs and the parallel evolution between dogs and humans. Nat Commun.

[8] Wang GD, Zhai W, Yang HC, Wang L, Zhong L, Liu YH, Fan RX, Yin TT, Zhu CL, Poyarkov AD (2016). Out of southern East Asia: the natural history of domestic dogs across the world. Cell Res.

[9] Vonholdt BM, Pollinger JP, Lohmueller KE, Han E, Parker HG, Quignon P, Degenhardt JD, Boyko AR, Earl DA, Auton A (2010). Genome-wide SNP and haplotype analyses reveal a rich history underlying dog domestication. Nature.

[10] Shannon LM, Boyko RH, Castelhano M, Corey E, Hayward JJ, McLean C, White ME, Abi Said M, Anita BA, Bondjengo NI (2015). Genetic structure in village dogs reveals a Central Asian domestication origin. Proc Natl Acad Sci U S A.

[11] Thalmann O, Shapiro B, Cui P, Schuenemann VJ, Sawyer SK, Greenfield DL, Germonpre MB, Sablin MV, Lopez-Giraldez F, Domingo-Roura X (2013). Complete mitochondrial genomes of ancient canids suggest a European origin of domestic dogs. Science.

[12] Lindblad-Toh K, Wade CM, Mikkelsen TS, Karlsson EK, Jaffe DB, Kamal M, Clamp M, Chang JL, Kulbokas EJ, Zody MC (2005). Genome sequence, comparative analysis and haplotype structure of the domestic dog. Nature.

[13] Club AK. The complete dog book. 20th ed. New York: Ballantine Books; 2006.

[14] Parker HG, Kim LV, Sutter NB, Carlson S, Lorentzen TD, Malek TB, Johnson GS, DeFrance HB, Ostrander EA, Kruglyak L (2004). Genetic structure of the purebred domestic dog. Science.

[15] Baird NA, Etter PD, Atwood TS, Currey MC, Shiver AL, Lewis ZA, Selker EU, Cresko WA, Johnson EA (2008). Rapid SNP discovery and genetic mapping using sequenced RAD markers. PLoS One.

[16] The Restriction Enzyme Database. http://rebase.neb.com/rebase/rebase.html. Accessed 8 Mar 2012.

[17] Auton A, Rui Li Y, Kidd J, Oliveira K, Nadel J, Holloway JK, Hayward JJ, Cohen PE, Greally JM, Wang J (2013). Genetic recombination is targeted towards gene promoter regions in dogs. PLoS Genet.

[18] Gou X, Wang Z, Li N, Qiu F, Xu Z, Yan D, Yang S, Jia J, Kong X, Wei Z (2014). Whole-genome sequencing of six dog breeds from continuous altitudes reveals adaptation to high-altitude hypoxia. Genome Res.

[19] Kim RN, Kim DS, Choi SH, Yoon BH, Kang A, Nam SH, Kim DW, Kim JJ, Ha JH, Toyoda A (2012). Genome analysis of the domestic dog (Korean Jindo) by massively parallel sequencing. DNA Res.

[20] von Holdt BM, Pollinger JP, Earl DA, Knowles JC, Boyko AR, Parker H, Geffen E, Pilot M, Jedrzejewski W, Jedrzejewska B (2011). A genome-wide perspective on the evolutionary history of enigmatic wolf-like canids. Genome Res.

[21] Li H, Durbin R (2010). Fast and accurate long-read alignment with Burrows-Wheeler transform. Bioinformatics.

[22] Picard. http://broadinstitute.github.io/picard.

[23] DePristo MA, Banks E, Poplin R, Garimella KV, Maguire JR, Hartl C, Philippakis AA, del Angel G, Rivas MA, Hanna M (2011). A framework for variation discovery and genotyping using next-generation DNA sequencing data. Nat Genet.

[24] Li H, Handsaker B, Wysoker A, Fennell T, Ruan J, Homer N, Marth G, Abecasis G, Durbin R, Genome Project Data Processing S (2009). The Sequence Alignment/Map format and SAMtools. Bioinformatics.

[25] Manichaikul A, Mychaleckyj JC, Rich SS, Daly K, Sale M, Chen WM (2010). Robust relationship inference in genome-wide association studies. Bioinformatics.

[26] Patterson N, Price AL, Reich D (2006). Population structure and eigenanalysis. PLoS Genet.

[27] Alexander DH, Novembre J, Lange K (2009). Fast model-based estimation of ancestry in unrelated individuals. Genome Res.

[28] Kopelman NM, Mayzel J, Jakobsson M, Rosenberg NA, Mayrose I (2015). Clumpak: a program for identifying clustering modes and packaging population structure inferences across K. Mol Ecol Resour.

[29] Purcell S, Neale B, Todd-Brown K, Thomas L, Ferreira MA, Bender D, Maller J, Sklar P, de Bakker PI, Daly MJ (2007). PLINK: a tool set for whole-genome association and population-based linkage analyses. Am J Hum Genet.

[30] American Kennel Club. http://www.akc.org/dog-breeds/chow-chow/detail/. Accessed 11 Nov 2015.

[31] Pickrell JK, Pritchard JK (2012). Inference of population splits and mixtures from genome-wide allele frequency data. PLoS Genet.

[32] Patterson N, Moorjani P, Luo Y, Mallick S, Rohland N, Zhan Y, Genschoreck T, Webster T, Reich D (2012). Ancient admixture in human history. Genetics.

[33] Gronau I, Hubisz MJ, Gulko B, Danko CG, Siepel A (2011). Bayesian inference of ancient human demography from individual genome sequences. Nat Genet.

[34] UCSC Genome Browser. http://hgdownload.soe.ucsc.edu/goldenPath/canFam3/database/. Accessed 8 July 2015.

[35] NCBI, GCF_000002285.3_CanFam3.1_genomic.gff.gz. ftp://ftp.ncbi.nlm.nih.gov/genomes/all/GCF/000/002/285/GCF_000002285.3_CanFam3.1/GCF_000002285.3_CanFam3.1_genomic.gff.gz. Accessed 8 July 2015.

[36] Freedman AH, Gronau I, Schweizer RM, Ortega-Del Vecchyo D, Han E, Silva PM, Galaverni M, Fan Z, Marx P, Lorente-Galdos B (2014). Genome sequencing highlights the dynamic early history of dogs. PLoS Genet.

[37] UCSC Genome Browser Ftp. ftp://hgdownload.cse.ucsc.edu/goldenPath/mm10/phastCons60way/euarchontoglire/. Accessed 14 July 2015.

[38] Kumar S, Subramanian S (2002). Mutation rates in mammalian genomes. Proc Natl Acad Sci U S A.

[39] Yi X, Liang Y, Huerta-Sanchez E, Jin X, Cuo ZX, Pool JE, Xu X, Jiang H, Vinckenbosch N, Korneliussen TS (2010). Sequencing of 50 human exomes reveals adaptation to high altitude. Science.

[40] Nielsen R, Williamson S, Kim Y, Hubisz MJ, Clark AG, Bustamante C (2005). Genomic scans for selective sweeps using SNP data. Genome Res.

[41] Danecek P, Auton A, Abecasis G, Albers CA, Banks E, DePristo MA, Handsaker RE, Lunter G, Marth GT, Sherry ST (2011). The variant call format and VCFtools. Bioinformatics.

[42] Flicek P, Amode MR, Barrell D, Beal K, Billis K, Brent S, Carvalho-Silva D, Clapham P, Coates G, Fitzgerald S (2014). Ensembl 2014. Nucleic Acids Res.

[43] da Huang W, Sherman BT, Lempicki RA (2009). Systematic and integrative analysis of large gene lists using DAVID bioinformatics resources. Nat Protoc.

[44] Bei M (2009). Molecular genetics of tooth development. Curr Opin Genet Dev.

[45] Kutmon M, Riutta A, Nunes N, Hanspers K, Willighagen EL, Bohler A, Melius J, Waagmeester A, Sinha SR, Miller R (2016). WikiPathways: capturing the full diversity of pathway knowledge. Nucleic Acids Res.

[46] Ornitz DM, Itoh N (2015). The Fibroblast Growth Factor signaling pathway. Wiley Interdiscip Rev Dev Biol.

[47] Montoliu L, Oetting WS, Bennett DC. Color Genes. http://www.espcr.org/micemut/. Accessed 3 Oct 2015.

[48] Hwang S, Kim E, Lee I, Marcotte EM (2015). Systematic comparison of variant calling pipelines using gold standard personal exome variants. Sci Rep.

[49] Oskarsson MC, Klutsch CF, Boonyaprakob U, Wilton A, Tanabe Y, Savolainen P (2012). Mitochondrial DNA data indicate an introduction through Mainland Southeast Asia for Australian dingoes and Polynesian domestic dogs. Proc Biol Sci.

[50] Lickert H, Takeuchi JK, Von Both I, Walls JR, McAuliffe F, Adamson SL, Henkelman RM, Wrana JL, Rossant J, Bruneau BG (2004). Baf60c is essential for function of BAF chromatin remodelling complexes in heart development. Nature.

[51] Albini S, Coutinho P, Malecova B, Giordani L, Savchenko A, Forcales SV, Puri PL (2013). Epigenetic reprogramming of human embryonic stem cells into skeletal muscle cells and generation of contractile myospheres. Cell Rep.

[52] Wan M, Wu X, Guan KL, Han M, Zhuang Y, Xu T (2006). Muscle atrophy in transgenic mice expressing a human TSC1 transgene. FEBS Lett.

[53] Selvaraj A, Prywes R (2003). Megakaryoblastic leukemia-1/2, a transcriptional co-activator of serum response factor, is required for skeletal myogenic differentiation. J Biol Chem.

[54] Flacco N, Segura V, Perez-Aso M, Estrada S, Seller JF, Jimenez-Altayo F, Noguera MA, D’Ocon P, Vila E, Ivorra MD (2013). Different beta-adrenoceptor subtypes coupling to cAMP or NO/cGMP pathways: implications in the relaxant response of rat conductance and resistance vessels. Br J Pharmacol.

[55] Wenstrup RJ, Florer JB, Brunskill EW, Bell SM, Chervoneva I, Birk DE (2004). Type V collagen controls the initiation of collagen fibril assembly. J Biol Chem.

[56] Wang X, Inoue S, Gu J, Miyoshi E, Noda K, Li W, Mizuno-Horikawa Y, Nakano M, Asahi M, Takahashi M (2005). Dysregulation of TGF-beta1 receptor activation leads to abnormal lung development and emphysema-like phenotype in core fucose-deficient mice. Proc Natl Acad Sci U S A.

[57] Montague MJ, Li G, Gandolfi B, Khan R, Aken BL, Searle SM, Minx P, Hillier LW, Koboldt DC, Davis BW (2014). Comparative analysis of the domestic cat genome reveals genetic signatures underlying feline biology and domestication. Proc Natl Acad Sci U S A.

[58] Rubin CJ, Zody MC, Eriksson J, Meadows JR, Sherwood E, Webster MT, Jiang L, Ingman M, Sharpe T, Ka S (2010). Whole-genome resequencing reveals loci under selection during chicken domestication. Nature.

[59] Kijas JW, Lenstra JA, Hayes B, Boitard S, Porto Neto LR, San Cristobal M, Servin B, McCulloch R, Whan V, Gietzen K (2012). Genome-wide analysis of the world’s sheep breeds reveals high levels of historic mixture and strong recent selection. PLoS Biol.

[60] Al-Shawi R, Ashton SV, Underwood C, Simons JP (2001). Expression of the Ror1 and Ror2 receptor tyrosine kinase genes during mouse development. Dev Genes Evol.

[61] Lin M, Li L, Liu C, Liu H, He F, Yan F, Zhang Y, Chen Y (2011). Wnt5a regulates growth, patterning, and odontoblast differentiation of developing mouse tooth. Dev Dyn.

[62] Zhang Z, Lan Y, Chai Y, Jiang R (2009). Antagonistic actions of Msx1 and Osr2 pattern mammalian teeth into a single row. Science.

[63] Zhao ZJ (2010). New data and new issues for the study of origin of rice agriculture in China. Archaeol Anthrop Sci.

[64] Feder JL, Egan SP, Nosil P (2012). The genomics of speciation-with-gene-flow. Trends Genet.

[65] Collins BJ, Deak M, Murray-Tait V, Storey KG, Alessi DR (2005). In vivo role of the phosphate groove of PDK1 defined by knockin mutation. J Cell Sci.

[66] Sulem P, Gudbjartsson DF, Stacey SN, Helgason A, Rafnar T, Jakobsdottir M, Steinberg S, Gudjonsson SA, Palsson A, Thorleifsson G (2008). Two newly identified genetic determinants of pigmentation in Europeans. Nat Genet.

[67] Norris BJ, Whan VA (2008). A gene duplication affecting expression of the ovine ASIP gene is responsible for white and black sheep. Genome Res.

[68] Drogemuller C, Giese A, Martins-Wess F, Wiedemann S, Andersson L, Brenig B, Fries R, Leeb T (2006). The mutation causing the black-and-tan pigmentation phenotype of Mangalitza pigs maps to the porcine ASIP locus but does not affect its coding sequence. Mamm Genome.

[69] Girardot M, Martin J, Guibert S, Leveziel H, Julien R, Oulmouden A (2005). Widespread expression of the bovine Agouti gene results from at least three alternative promoters. Pigment Cell Res.

[70] Rieder S, Taourit S, Mariat D, Langlois B, Guerin G (2001). Mutations in the agouti (ASIP), the extension (MC1R), and the brown (TYRP1) loci and their association to coat color phenotypes in horses (Equus caballus). Mamm Genome.

[71] Schubert M, Jonsson H, Chang D, Der Sarkissian C, Ermini L, Ginolhac A, Albrechtsen A, Dupanloup I, Foucal A, Petersen B (2014). Prehistoric genomes reveal the genetic foundation and cost of horse domestication. Proc Natl Acad Sci U S A.

