# Supplementary material for: The origin of chow chows in the light of the East Asian breeds
Source: BMC Genomics. 2017 Feb 16;18:174. doi: 10.1186/s12864-017-3525-9 (PMC5312535; doi:10.1186/s12864-017-3525-9)
Supplement: Additional file 2: Note S1. — Mutation Rate Estimation. (DOCX 44 kb) [file 12864_2017_3525_MOESM2_ESM.docx]

**Supplementary Notes**

**Note S1 Mutation Rate Estimation**

Mutation rate is a fundamental parameter in demographic inference. There are two mutation rates commonly used in the studies of dogs. One is 1.0*10^-8^ per site per generation [[1-3](#_ENREF_1)] and the other is 6.6*10^-9^ per site per generation (i.e. 2.2*10^-9^ per site per year) assuming generation time of three years [[4](#_ENREF_4), [5](#_ENREF_5)]. From literature review, we found that the origin of 1.0*10^-8^ is a simulation study conducted in the original dog genome work trying to simulate under a variety of demographic scenarios [[Supplementary Table S20 1](#_ENREF_1)]. We didn’t find a further source of this rate and thus think this might be casual adoption of a mutation rate commonly used in human population genetics.

In order to get the true history of dogs, we used Compara data from Ensembl to estimate the mutation rate along dog lineage. Although there are 39 species in this data set, only 17 of them are the core species and have good genome coverage and assembly (the rest of the species are only sequenced at around 2X coverage). We focus the analysis on the core species to avoid confounding effects from assembly/sequencing error ([ftp://ftp.ensembl.org/pub/release-79/maf/ensembl- compara/multiple_alignments/epo_17_eutherian](ftp://ftp.ensembl.org/pub/release-79/maf/ensembl-%20compara/multiple_alignments/epo_17_eutherian), downloaded on May 13, 2015).

Using human as the outgroup and cat, horse, cattle, sheep, pig as sister species to dog, we estimated branch length leading to dogs in phylogenetic tree ((dog; sister_species); human). In order to extract putatively neutral sequences, we downloaded the annotation data from Ensembl ([ftp://ftp.ensembl.org/pub /release-79/gtf](ftp://ftp.ensembl.org/pub%20/release-79/gtf), downloaded on May 13, 2015) for those Genomes and used MafFilter [[6](#_ENREF_6)] to extracted sequences outside of gene regions in any species of each phylogenetic tree. Using the baseml program from the PAML package [[7](#_ENREF_7)], we estimate the branch length leading to the dog lineage (assuming a general time reversible (GTR) model and gamma distributed rate variation).

Using the evolutionary distances estimated from the baseml together with the divergence time from the literature [[8](#_ENREF_8)], we can estimate the mutation rate along the dog lineage at different levels of divergence. From the results shown in Table S5, we can find that the mutation rate on the lineage leading to dog is increasing, and is about 2.13*10^-9^ per base per year after it diverged from cat. So we used 2.2*10^-9^ per year as the mutation rate of dogs in this study, a rate also found in several other studies [[5](#_ENREF_5), [9](#_ENREF_9)].

**References**

1. Lindblad-Toh K, Wade CM, Mikkelsen TS, Karlsson EK, Jaffe DB, Kamal M, Clamp M, Chang JL, Kulbokas EJ, 3rd, Zody MC *et al*: **Genome sequence, comparative analysis and haplotype structure of the domestic dog**. *Nature* 2005, **438**(7069):803-819.

2. Freedman AH, Gronau I, Schweizer RM, Ortega-Del Vecchyo D, Han E, Silva PM, Galaverni M, Fan Z, Marx P, Lorente-Galdos B *et al*: **Genome sequencing highlights the dynamic early history of dogs**. *PLoS Genet* 2014, **10**(1):e1004016.

3. Gray MM, Granka JM, Bustamante CD, Sutter NB, Boyko AR, Zhu L, Ostrander Ea, Wayne RK: **Linkage disequilibrium and demographic history of wild and domestic canids.** *Genetics* 2009, **181**:1493-1505.

4. Wang GD, Zhai W, Yang HC, Fan RX, Cao X, Zhong L, Wang L, Liu F, Wu H, Cheng LG *et al*: **The genomics of selection in dogs and the parallel evolution between dogs and humans**. *Nat Commun* 2013, **4**:1860.

5. Liu GE, Matukumalli LK, Sonstegard TS, Shade LL, Van Tassell CP: **Genomic divergences among cattle, dog and human estimated from large-scale alignments of genomic sequences**. *BMC Genomics* 2006, **7**.

6. Dutheil JY, Gaillard S, Stukenbrock EH: **MafFilter: a highly flexible and extensible multiple genome alignment files processor**. *BMC Genomics* 2014, **15**.

7. Yang ZH: **PAML 4: Phylogenetic analysis by maximum likelihood**. *Mol Biol Evol* 2007, **24**(8):1586-1591.

8. Hedges SB: **The origin and evolution of model organisms**. *Nat Rev Genet* 2002, **3**(11):838-849.

9. Kumar S, Subramanian S: **Mutation rates in mammalian genomes**. *Proc Natl Acad Sci U S A* 2002, **99**(2):803-808.
