# Supplementary figures and images for: The origin of chow chows in the light of the East Asian breeds
Source: BMC Genomics. 2017 Feb 16;18:174. doi: 10.1186/s12864-017-3525-9 (PMC5312535; doi:10.1186/s12864-017-3525-9)

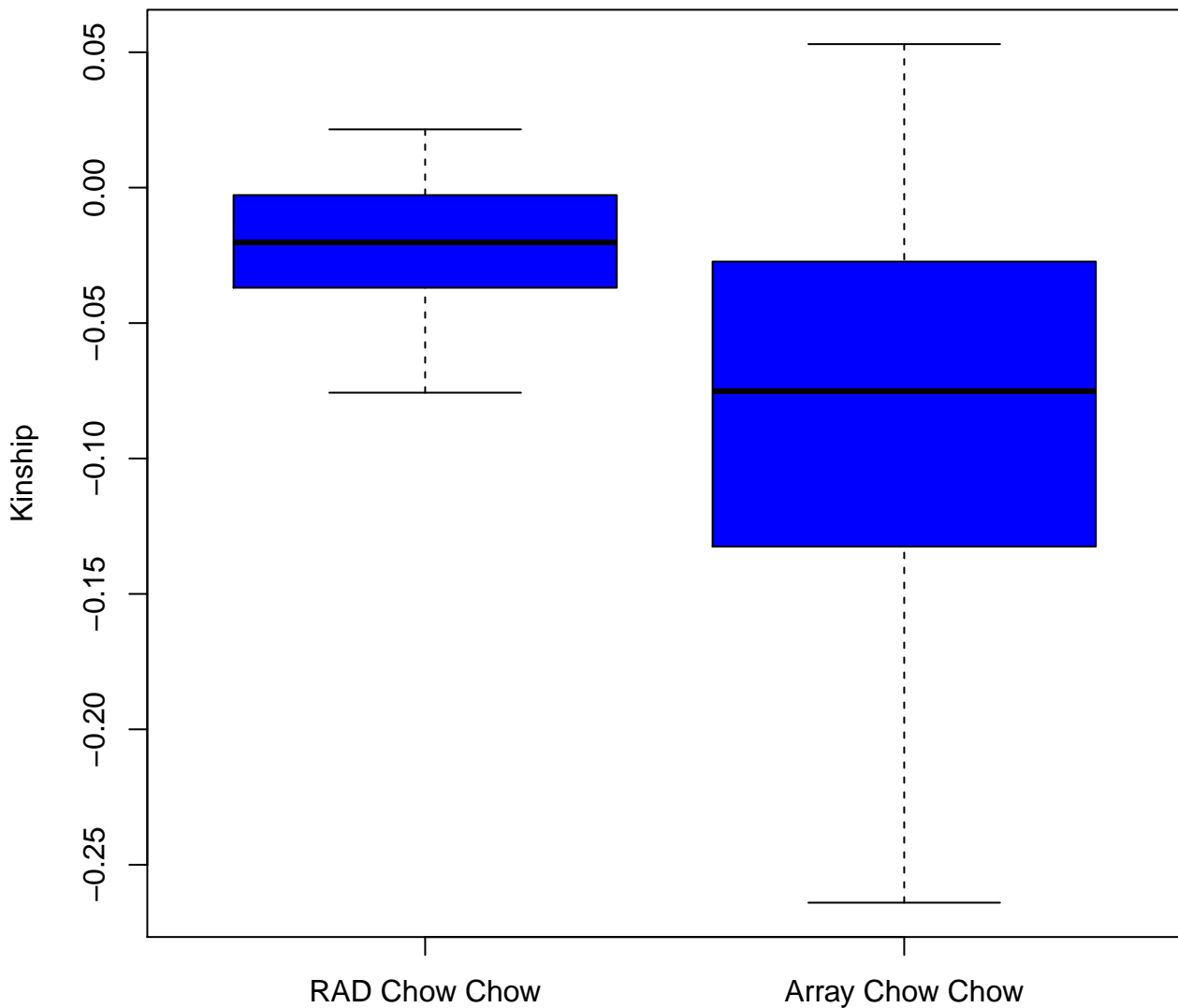

Supplement: Additional file 3: Figure S1. — Boxplot of the kinship coefficient between pairs of Chow Chows in the RAD set and from the SNP array data. Different levels of relatedness will yield different kinship coefficients. For example, it is suggested that the estimated kinship coefficient range [0.354, 1], [0.177, 0.354], [0.0884, 0.177] and [0.0442, 0.0884] correspond to duplicate/monozygotic twin, 1st-degree, 2nd-degree, and 3rd-degree relationships respectively. All kinship coefficients from the RAD sequenced Chow Chows are smaller than 0.0442, and are comparable to the Chow Chows from the SNP array data. (PDF 4 kb) [file 12864_2017_3525_MOESM3_ESM.pdf]

A

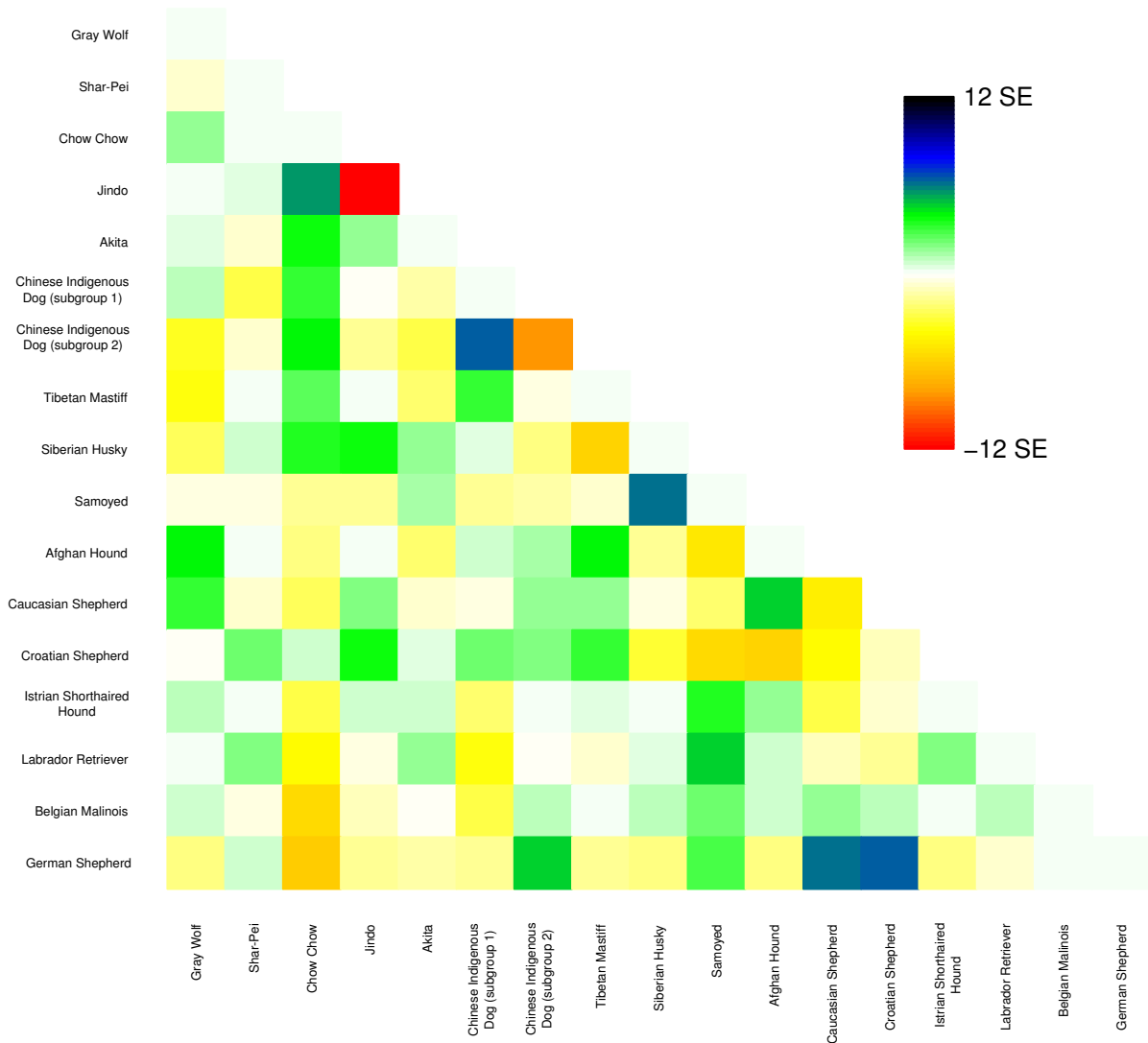

**B**

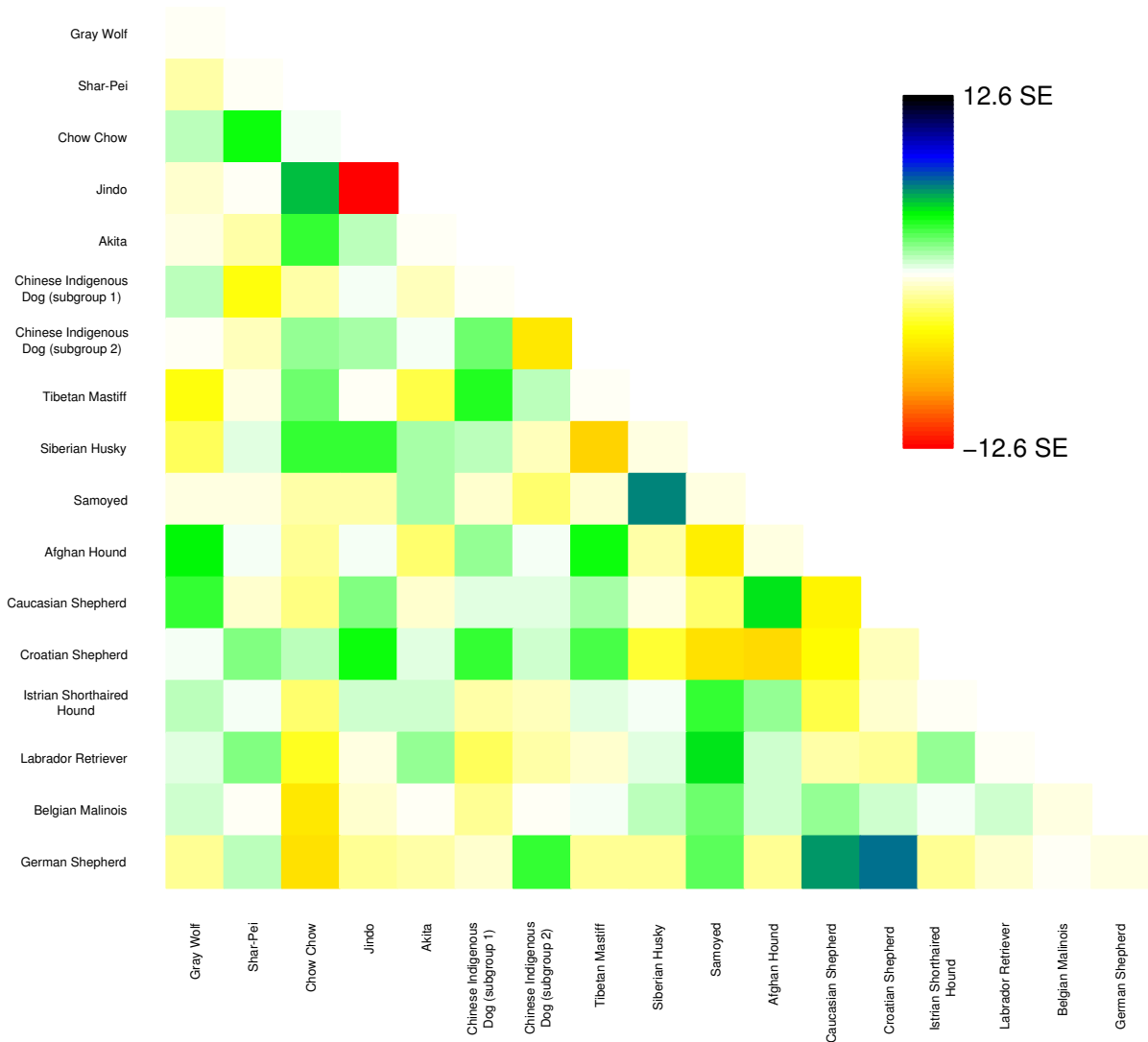

Supplement: Additional file 4: Figure S2. — Residual of the TreeMix analysis presented in Fig. 4. Panel A and B correspond to the panels in the Fig. 4. (PDF 116 kb) [file 12864_2017_3525_MOESM4_ESM.pdf]
